# Treatment Satisfaction and Well-Being With Continuous Glucose Monitoring in People With Type 1 Diabetes: An Analysis Based on the GOLD Randomized Trial

**DOI:** 10.1177/19322968231183974

**Published:** 2023-07-27

**Authors:** Daniel Pylov, William Polonsky, Henrik Imberg, Helen Holmer, Jarl Hellman, Magnus Wijkman, Jan Bolinder, Tim Heisse, Sofia Dahlqvist, Thomas Nyström, Erik Schwarz, Irl Hirsch, Marcus Lind

**Affiliations:** 1 Department of Molecular and Clinical Medicine, Sahlgrenska Academy, University of Gothenburg, Gothenburg, Sweden; 2 Department of Medicine, Sahlgrenska University Hospital, Gothenburg, Sweden; 3 Behavioral Diabetes Institute, San Diego, CA, USA; 4 University of California, San Diego, CA, USA; 5 Chalmers University of Technology, Gothenburg, Sweden; 6 University of Gothenburg, Gothenburg, Sweden; 7 Statistiska Konsultgruppen, Gothenburg, Sweden; 8 Department of Internal Medicine, Centralsjukhuset, Kristianstad, Sweden; 9 Department of Medical Sciences, Clinical Diabetes and Metabolism, Uppsala University, Uppsala, Sweden; 10 Department of Internal Medicine and Department of Health, Medicine and Caring Sciences, Linköping University, Norrköping, Sweden; 11 Department of Medicine, Karolinska University Hospital Huddinge, Karolinska Institutet, Stockholm, Sweden; 12 Profil, Neuss, Germany; 13 Department of Medicine, NU Hospital Group, Uddevalla, Sweden; 14 Department of Clinical Science and Education, Södersjukhuset, Karolinska Institutet, Stockholm, Sweden; 15 Department of Internal Medicine, Faculty of Medicine & Health, Örebro University, Örebro Sweden; 16 School of Medicine, University of Washington, Seattle, WA, USA

**Keywords:** CGMS, MDI, quality of life, SMBG, treatment satisfaction, type 1 diabetes

## Abstract

**Background:**

The GOLD trial demonstrated that continuous glucose monitoring (CGM) in people with type 1 diabetes (T1D) managed with multiple daily insulin injections (MDI) improved not only glucose control but also overall well-being and treatment satisfaction. This analysis investigated which factors contributed to improved well-being and treatment satisfaction with CGM.

**Methods:**

The GOLD trial was a randomized crossover trial comparing CGM versus self-monitored blood glucose (SMBG) over 16 months. Endpoints included well-being measured by the World Health Organization–Five Well-Being Index (WHO-5) and treatment satisfaction by the Diabetes Treatment Satisfaction Questionnaire (DTSQ) as well as glucose metrics. Multivariable R^2^-decomposition was used to understand which variables contributed most to treatment satisfaction.

**Results:**

A total of 139 participants were included. Multivariable analyses revealed that increased convenience and flexibility contributed to 60% (95% confidence interval [CI] = 50%-69%) of the improvement in treatment satisfaction (Diabetes Treatment Satisfaction Questionnaire *change* version [DTSQ*c*]) observed with CGM, whereas perceived effects on hypoglycemia and hyperglycemia only contributed to 6% (95% CI = 2%-11%) of improvements. Significant improvements in well-being (WHO-5) by CGM were observed for the following: feeling cheerful (*P* = .025), calm and relaxed (*P* = .024), being active (*P* = .046), and waking up fresh and rested (*P* = .044). HbA1c reductions and increased time in range (TIR) were associated with increased treatment satisfaction, whereas glycemic variability was not. HbA1c reduction showed also an association with increased well-being and increased TIR with less diabetes-related distress.

**Conclusions:**

While CGM improves glucose control in people with T1D on MDI, increased convenience and flexibility through CGM is of even greater importance for treatment satisfaction and patient well-being. These CGM-mediated effects should be taken into account when considering CGM initiation.

## Introduction

Type 1 diabetes mellitus (T1D) is a disease that requires constant monitoring of blood glucose levels and daily diabetes management, 24/7, 365 days a year. It is a long-term health condition that constantly carries an emotional, physical, and financial burden, even at times when the disease itself does not bother the patient.^1^ Many patients feel overwhelmed and frustrated by the influence of T1D, and as a result it can lead to depression, anxiety, and worse diabetes outcomes.^2^ The prevalence rate of depression is more than three times higher in people with T1D compared with those without.^3^ In clinical practice, the quality of diabetes management is evaluated by glucose metrics, including HbA1c, time in range (TIR), time above range (TAR), time below range (TBR) and glucose variability, but generally not treatment satisfaction and well-being. Advanced diabetes technologies, including continuous glucose monitoring (CGM) systems, remain less accessible in many countries.^4^ Over time, CGM has become more common in developed nations, but self-monitoring of blood glucose (SMBG) is still the most common method in wide geographic regions such as Africa, South America, Asia, and Eastern Europe. Therefore, increased understanding of the potential benefits of CGM versus SMBG is of importance. One limitation to more widespread availability of glucose monitoring methods has been its cost, often more expensive than insulin therapy per se.^5-7^

In creating a plan to manage T1D, clinicians should not only take into account how treatment affects the “numbers” but also patient well-being. Previously in the GOLD trial, CGM was shown to have beneficial effects on well-being and treatment satisfaction,^8^ but factors leading to these effects have not been studied. The current analysis investigated factors contributing to improved well-being and treatment satisfaction with CGM in persons with T1D managed with multiple daily insulin injections (MDI).

## Material and Methods

The design of the GOLD trial has been described in detail and was approved by the ethics committee of the University of Gothenburg, Gothenburg, Sweden.^9^All participants gave verbal and written informed consent. Briefly, the GOLD trial was a randomized, open-label, controlled trial with a crossover design conducted over 69 weeks. Of 161 individuals randomized, 141 with available HbA1c values and continuing treatment throughout the study in both treatment phases were included in the primary analysis.^8^ After a run-in period of up to eight weeks, patients were followed for 69 weeks where each treatment period lasted 26 weeks with a washout period of 17 weeks in between. Patients were randomized 1:1 to glucose monitoring using a CGM (Dexcom G4) stand-alone system or capillary SMBG. Enrolled patients were given basic instruction on insulin dosing, bolus correction, meals that can influence glucose levels, and effects of physical activity on glucose control.

Masked CGM was performed in two of the last four weeks when participants used SMBG for glucose monitoring to compare CGM metrics between the two treatment sequences. To evaluate treatment experience, participants completed a series of self-reported questionnaires before and after each treatment phase, including

1. Diabetes Treatment Satisfaction Questionnaire *status* version (DTSQ*s*)—original eight-item “status” form.^10^
2. World Health Organization–Five Well-Being Index (WHO-5) survey questionnaire—five-item scale used for collecting and assessing data related to patient well-being.^11^
3. Problem Areas in Diabetes Questionnaire, Swedish version (Swe-PAID-20)—a validated questionnaire with a 20-item scale regarding emotional distress in patients with diabetes.^1,12,13^

At the end of the study (week 69), patients completed the Diabetes Treatment Satisfaction Questionnaire *change* version (DTSQ*c*), an eight-item scale where patients compare treatment experience and satisfaction (intraindividual comparisons).^14^

### Procedures

To evaluate effects on treatment satisfaction, well-being, and emotional distress, comparisons of the DTSQ*s*, WHO-5, and Swe-PAID-20 questionnaires were performed at the end of each treatment phase. Each individual item from each questionnaire was compared between CGM and SMBG sequences to understand what fields explained differences in treatment satisfaction, well-being, and emotional distress between CGM and SMBG. Corresponding analyses were performed for the DTSQ*c* relating each item to the type of glucose monitoring method (CGM vs SMBG).

To understand whether changes in certain glucose metrics were specifically related to treatment satisfaction, well-being, or emotional distress, we correlated changes in DTSQ*s*, WHO-5, and Swe-PAID-20 scores to changes in various glucose metrics between CGM and SMBG treatment sequences. Corresponding analyses were performed for the DTSQ*c*. HbA1c and the following CGM metrics were evaluated: mean glucose level, time in euglycemia, TIR, TAR, TBR, coefficient of variation (CV), standard deviation (SD), and mean amplitude of glycemic excursions (MAGE). Euglycemia, TIR, TAR, and TBR were analyzed for the following categories in mmol/L (mg/dL): TBR <3.0 (54), TBR <3.9 (70), time in euglycemia (3.9-8.0 (70-144), TIR 3.9–10 (70-180), TAR >10 (180), and TAR >13.9 (250).

We also evaluated whether certain patient groups benefited more or less from CGM with respect to treatment satisfaction, well-being, and emotional distress by correlating baseline patient characteristics with changes in the overall scores of the questionnaires between CGM and SMBG treatment phases. The following baseline characteristics were considered: age, sex, HbA1c, diabetes duration, number of hypoglycemia events, time in euglycemia, TIR, TAR, and TBR.

### Statistics

For descriptive purposes, data are presented as mean, standard deviation (SD), median, minimum, and maximum value for numeric variables, and as number and percent for categorical variables. Differences between groups were analyzed using the sign test on matched ordinal data by comparing the number of patients for whom the outcome increased or decreased between treatment phases. Correlation analyses were performed using Pearson correlation coefficients (*r*), presented with 95% confidence intervals (CIs).

To quantify the relative importance of each DTSQ*c* item to overall treatment satisfaction with CGM compared with SMBG, multivariable R^2^-decomposition was performed using the LMG method.^15,16^ Compared with the effect size or raw magnitude of differences in individual items, this gives a simple summary of the effect as the fraction of the total variance in treatment satisfaction (DTSQ*c* item 1) that may be attributed by each of the other DTSQ*c* items while accounting for correlations between items. Confidence intervals were calculated using nonparametric bootstrap with 1000 bootstrap replicates.

All statistical tests were two-tailed and conducted at the 5% significance level. Statistical analyses were performed using SAS Software version 9.4 (SAS Institute, Cary, North Carolina) and R language and environment for statistical computing version 4.1.3 (R Core Team, Vienna, Austria). Multivariable R^2^-decomposition was performed using the R relaimpo version 2.2-6 package.

## Results

### Patient Characteristics

A total of 139 study participants had information from the questionnaires in both treatment phases and were included in the current analysis. Mean (SD) age at baseline was 44.6 (12.6) years, 61 (43.9%) were women, mean duration of diabetes was 22.1 (11.9) years, and mean HbA1c was 8.7% (0.8) or 71.8 mmol/mol (9.1). Overall patient characteristics were similar to all randomized patients (n = 161) (Supplemental Table S1).

#### Treatment satisfaction

There was a significant difference in overall treatment satisfaction with CGM compared with SMBG according to DTSQ*c* (*P* < .001), with 83 (61.0%) patients reporting a preference for CGM compared with 43 (31.6%) preferring SMBG (Table 1). Patients also reported a reduction in perceived time with unacceptably high blood glucose levels with CGM (*P* = .013). There were no significant differences with respect to perceived time in unacceptably low blood glucose levels (*P* = .20) and satisfaction in understanding diabetes (*P* = .19). DTSQ*s* results showed an overall similar pattern as DTSQ*c* (Supplemental Table S2).

**Table 1.** Comparison of DTSQ*c* Between CGM and SMBG Treatments.

DTSQ*c* item	CGM vs SBMG (n = 136)	*P* value
Q1: How satisfied are you with your current treatment?		
Less satisfied now (−3 to −1)	43 (31.6%)	
Equally satisfied now (0)	10 (7.4%)	
More satisfied now (1 to 3)	83 (61.0%)	*P* < .001
Q2: How often have you felt that your blood sugars have been unacceptably high recently?		
Less of the time now (−3 to −1)	74 (54.8%)	
Equally of the time now (0)	15 (11.1%)	
More of the time now (1 to 3)	46 (34.1%)	*P* = .013
Q3: How often have you felt that your blood sugars have been unacceptably low recently?		
Less of the time now (−3 to −1)	58 (43.0%)	
Equally of the time now (0)	33 (24.4%)	
More of the time now (1 to 3)	44 (32.6%)	*P* = .20
Q4: How convenient have you been finding your treatment to be recently?		
Less convenient now (−3 to −1)	44 (32.6%)	
Equally convenient now (0)	13 (9.6%)	
More convenient now (1 to 3)	78 (57.8%)	*P* = .003
Q5: How flexible have you been finding your treatment to be recently?		
Less flexible now (−3 to −1)	38 (27.9%)	
Equally flexible now (0)	20 (14.7%)	
More flexible now (1 to 3)	78 (57.4%)	*P* < .001
Q6: How satisfied are you with your understanding of your diabetes?		
Less satisfied now (−3 to −1)	49 (36.3%)	
Equally satisfied now (0)	22 (16.3%)	
More satisfied now (1 to 3)	64 (47.4%)	*P* = .19
Q7: Would you recommend this form of treatment to someone else with your kind of diabetes?		
Less likely to recommend the treatment now (−3 to −1)	49 (36.0%)	
Equally likely to recommend the treatment now (0)	10 (7.4%)	
More likely to recommend the treatment now (1 to 3)	77 (56.6%)	*P* = .016
Q8: How satisfied would you be to continue with your present form of treatment?		
Less satisfied now (−3 to −1)	44 (32.4%)	
Equally satisfied now (0)	11 (8.1%)	
More satisfied now (1 to 3)	81 (59.6%)	*P* = .001

Data are presented as number (percent). Each item was scored on a scale from −3 (much less satisfied now) to 3 (much more satisfied now) and presented in categories from −3 to −1 (less satisfied now), 0 (equally satisfied now), and 1 to 3 (more satisfied now). A positive value means preference for CGM. Change data are presented as number and percentage with decrease, equal, or increase in scores between treatments. Comparisons between treatments were performed using the sign test.

Abbreviations: CGM, continuous glucose monitoring; DTSQ*c*, Diabetes Treatment Satisfaction Questionnaire *change* version; Q, question/item; SMBG, self-monitoring of blood glucose.

**Figure 1.**
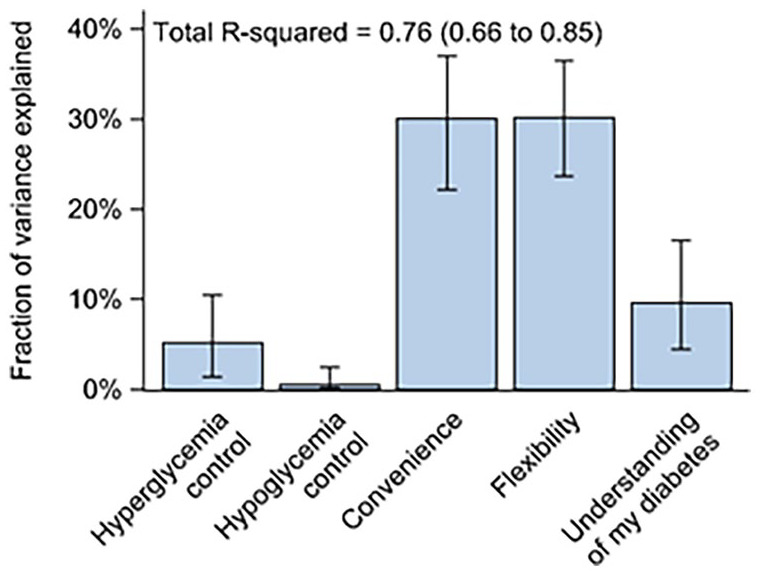
Fraction of total variance in satisfaction of CGM versus SMBG (DTSQ*c* item 1) explained by patient-reported hyperglycemia and hypoglycemia control, convenience, flexibility, and understanding of diabetes (DTSQ*c* items 2-6). Error bars represent 95% CIs. Analyses were performed using multivariable R2-decomposition. Abbreviations: CGM, continuous glucose monitoring; CI, confidence interval; DTSQ*c*, Diabetes Treatment Satisfaction Questionnaire *change* version; SMBG, self-monitored blood glucose.

Multivariable analyses showed that 76% (95% CI = 66%-85%) of the variation in overall treatment satisfaction (DTSQ*c* item 1) could be explained by patient perceptions of hyperglycemia and hypoglycemia control, convenience, flexibility, and diabetes understanding (DTSQ*c* items 2-6) (Figure 1). The most important factors in treatment satisfaction were increased convenience and flexibility, each of which explained 30% of the variation in treatment satisfaction (95% CI = 22%-37% and 24%-36%, respectively). Perceived hypoglycemia and hyperglycemia control explained only 6% of the total variation in treatment satisfaction (Figure 1).

#### Well-being and T1D management

Data from the WHO-5 questionnaire showed that patients experienced a significant increase in feeling cheerful and in good spirits (*P* = .025), feeling calm and relaxed (*P* = .024), feeling active and vigorous (*P* = .046), and quality of rest (*P* = .044) with CGM compared with SMBG (Table 2). There was no significant difference with respect to the last item, “My daily life has been filled with things that interest me” (*P* = .17).

**Table 2.** Comparison of the WHO-5 Measure of Current Mental Well-Being Between CGM and SMBG Treatments.

WHO-5 item	CGM (n = 137)	SMBG (n = 137)	Change from SMBG to CGM	*P* value
Q1: I have felt cheerful and in good spirits	3.6 (1.0) 4 (0; 5)	3.5 (0.9) 4 (1; 5)	Decrease 27 (19.7%) Equal 67 (48.9%) Increase 43 (31.4%)	*P* = .025
Q2: I have felt calm and relaxed	3.4 (1.1) 4 (0; 5)	3.2 (1.1) 3 (1; 5)	Decrease 32 (23.4%) Equal 58 (42.3%) Increase 47 (34.3%)	*P* = .024
Q3: I have felt active and vigorous	3.2 (1.2) 3 (0; 5)	3.0 (1.0) 3 (0; 5)	Decrease 31 (22.6%) Equal 51 (37.2%) Increase 55 (40.1%)	*P* = .046
Q4: I woke up feeling fresh and rested	2.8 (1.3) 3 (0; 5)	2.6 (1.2) 3 (0; 5)	Decrease 34 (24.8%) Equal 46 (33.6%) Increase 57 (41.6%)	*P* = .044
Q5: My daily life has been filled with things that interest me	3.6 (1.2) 4 (0; 5)	3.5 (1.0) 4 (0; 5)	Decrease 30 (21.9%) Equal 61 (44.5%) Increase 46 (33.6%)	*P* = .17

Data within groups are presented as mean (SD) and median (minimum value; maximum value). 0 = none of the time, 5 = all of the time. Change data are presented as number and percentage with decrease, equal or increase in scores between treatments. Comparisons between treatments were performed using the sign test.

Abbreviations: CGM, continuous glucose monitoring; Q, question/item; SMBG, self-monitoring of blood glucose; WHO-5, World Health Organization–Five Well-Being Index.

#### Problem areas in diabetes

Results from the Swe-PAID-20 questionnaire are presented in Table 3. During CGM treatment, there was a lower score for feeling discouraged with the diabetes treatment plan compared with SMBG (*P* = .008), feeling depressed when thinking about living with diabetes (*P* = .010), not knowing if mood or feelings are related to diabetes (*P* = .016), and feelings of guilt or anxiety when getting off track with diabetes management (*P* = .028). No other items of the Swe-PAID-20 questionnaire differed significantly between treatments.

**Table 3.** Comparison of Swe-PAID-20 Questionnaire Between CGM and SMBG Treatments.

Swe-PAID-20 item	CGM (n = 139)	SMBG (n = 139)	Change from SMBG to CGM	*P* value
Q1: Not having clear and concrete goals for your diabetes care?	1.1 (1.0) 1 (0; 3)	1.2 (1.0) 1 (0; 4)	Decrease 43 (31.2%) Equal 61 (44.2%) Increase 34 (24.6%)	*P* = .11
Q2: Feeling discouraged with your diabetes treatment plan?	0.8 (0.9) 1 (0; 4)	1.1 (1.0) 1 (0; 4)	Decrease 42 (30.4%) Equal 73 (52.9%) Increase 23 (16.7%)	*P* = .008
Q3: Feeling scared when you think about living with diabetes?	1.1 (1.0) 1 (0; 4)	1.1 (1.2) 1 (0; 4)	Decrease 31 (22.3%) Equal 76 (54.7%) Increase 32 (23.0%)	*P* = 0.55
Q4: Uncomfortable social situations related to your diabetes care?	0.9 (1.0) 1 (0; 4)	0.8 (1.1) 1 (0; 4)	Decrease 23 (17.2%) Equal 75 (56.0%) Increase 36 (26.9%)	*P* = .35
Q5: Feelings of deprivation regarding food and meals?	0.8 (0.8) 1 (0; 3)	0.8 (0.9) 1 (0; 3)	Decrease 28 (20.7%) Equal 79 (58.5%) Increase 28 (20.7%)	*P* = 1.00
Q6: Feeling depressed when you think about living with diabetes?	1.0 (1.0) 1 (0; 4)	1.2 (1.1) 1 (0; 4)	Decrease 41 (29.5%) Equal 74 (53.2%) Increase 24 (17.3%)	*P* = .010
Q7: Not knowing if your mood or feelings are related to your diabetes?	1.0 (0.9) 1 (0; 4)	1.2 (1.0) 1 (0; 4)	Decrease 38 (27.5%) Equal 76 (55.1%) Increase 24 (17.4%)	*P* = .016
Q8: Feeling overwhelmed by your diabetes?	1.2 (0.9) 1 (0; 4)	1.2 (1.1) 1 (0; 4)	Decrease 43 (31.2%) Equal 62 (44.9%) Increase 33 (23.9%)	*P* = .26
Q9: Worrying about low blood sugar reactions?	1.1 (1.0) 1 (0; 4)	1.2 (1.0) 1 (0; 4)	Decrease 36 (25.9%) Equal 73 (52.5%) Increase 30 (21.6%)	*P* = .74
Q10: Feeling angry when you think about living with diabetes?	0.9 (1.0) 1 (0; 4)	0.8 (1.0) 1 (0; 4)	Decrease 29 (20.9%) Equal 75 (54.0%) Increase 35 (25.2%)	*P* = .26
Q11: Feeling constantly concerned about food and eating?	1.0 (0.9) 1 (0; 3)	1.0 (1.1) 1 (0; 4)	Decrease 35 (25.2%) Equal 71 (51.1%) Increase 33 (23.7%)	*P* = .52
Q12: Worrying about the future and the possibility of serious complications?	1.8 (1.1) 2 (0; 4)	1.9 (1.1) 2 (0; 4)	Decrease 38 (27.3%) Equal 67 (48.2%) Increase 34 (24.5%)	*P* = .45
Q13: Feelings of guilt or anxiety when you get off track with your diabetes management?	1.3 (1.0) 1 (0;4)	1.5 (1.2) 1 (0; 4)	Decrease 44 (31.7%) Equal 69 (49.6%) Increase 26 (18.7%)	*P* = .028
Q14: Not accepting your diabetes?	0.6 (0.9) 0 (0; 4)	0.7 (1.0) 0 (0; 4)	Decrease 25 (18.0%) Equal 94 (67.6%) Increase 20 (14.4%)	*P* = .22
Q15: Feeling unsatisfied with your diabetes physician?	0.2 (0.5) 0 (0; 2)	0.3 (0.6) 0 (0; 3)	Decrease 19 (13.7%) Equal 105 (75.5%) Increase 15 (10.8%)	*P* = .70
Q16: Feeling that diabetes is taking up too much of your mental and physical energy every day?	1.0 (1.0) 1 (0; 4)	1.1 (1.1) 1 (0; 4)	Decrease 33 (23.7%) Equal 77 (55.4%) Increase 29 (20.9%)	*P* = .18
Q17: Feeling alone with your diabetes?	0.6 (0.8) 0 (0; 3)	0.7 (0.9) 0 (0; 4)	Decrease 31 (22.3%) Equal 78 (56.1%) Increase 30 (21.6%)	*P* = .91
Q18: Feeling that your friends and family are not supportive of your diabetes management efforts?	0.4 (0.7) 0 (0; 3)	0.5 (0.7) 0 (0; 3)	Decrease 20 (14.4%) Equal 104 (74.8%) Increase 15 (10.8%)	*P* = .21
Q19: Coping with complications of diabetes?	0.9 (1.0) 1 (0; 4)	0.9 (1.1) 1 (0; 4)	Decrease 29 (20.9%) Equal 85 (61.2%) Increase 25 (18.0%)	*P* = .56
Q20: Feeling unsatisfied with your diabetes specialist nurse?	0.1 (0.3) 0 (0; 2)	0.1 (0.5) 0 (0; 4)	Decrease 10 (7.2%) Equal 125 (89.9%) Increase 4 (2.9%)	*P* = .10

Data within groups are presented as mean (SD) and median (minimum value; maximum value). 0 = not a problem, 4 = serious problem. Change data are presented as number and percentage with decrease, equal or increase in scores between treatments. Comparisons between treatments were performed using the sign test.

Abbreviations: Swe-PAID-20, Problem Areas in Diabetes Questionnaire, Swedish version; CGM, continuous glucose monitoring; Q, question/item; SMBG, self-monitoring of blood glucose.

#### Correlation with changes in glycemic metrics

A lower reduction in HbA1c with CGM compared with SMBG was associated with lower increase in treatment satisfaction and well-being with CGM according to the DTSQ*s* (*r* = −0.23, *P* = .016) and WHO-5 (*r* = −0.20, *P* = .028) (Table 4). A greater increase in time in euglycemia (3.9–8 mmol/L and TIR = 3.9–10 mmol/L) during CGM treatment compared with SMBG was positively correlated with change in DTSQ*c* (*r* = 0.20, *P* = .027; *r* = 0.27, *P* = .003, respectively) and negatively correlated with change in Swe-PAID-20 (*r* = −0.20, *P* = .027; *r* = −0.22, *P* = .017, respectively). More time in hyperglycemia was associated with greater diabetes problems according to Swe-PAID-20 (*r* = 0.19, *P* = .040 for time >10 mmol/L) and less treatment satisfaction according to DTSQ*c* (*r* = −0.19, *P* = .039 for time >13.9 mmol/L). There were no significant associations between change in glycemic variability measures, CGM SD, CV, and MAGE, with change in treatment satisfaction and well-being with CGM compared with SMBG (Table 4).

**Table 4.** Correlation Between Change in Glycemic Outcome Measures From SMBG to CGM Treatment With Change in Diabetes Treatment Satisfaction and Quality of Life From SBMG to CGM Treatment.

	Pearson correlation coefficient (95% CI)			
	DTSQ*s*	DTSQ*c*	WHO-5	Swe-PAID-20
CGM SD	−0.11 (−0.29 to 0.08) *P* = .26	−0.06 (−0.24 to 0.12) *P* = .52	−0.16 (−0.33 to 0.02) *P* = .076	−0.01 (−0.19 to 0.17) *P* = .94
CGM CV	0.04 (−0.14 to 0.23) *P* = .65	0.11 (−0.08 to 0.28) *P* = .25	−0.03 (−0.21 to 0.15) *P* = .76	−0.01 (−0.19 to 0.17) *P* = .93
HbA1c (mmol/mol)	**−0.23 (−0.40 to −0.04)** *****P*** = .016**	−0.14 (−0.31 to 0.05) *P* = .14	**−0.20 (−0.37 to −0.02)** ***P*** **= .028**	0.02 (−0.16 to 0.19) *P* = .87
MAGE	−0.09 (−0.27 to 0.09) *P* = .32	−0.12 (−0.29 to 0.06) *P* = .20	−0.09 (−0.27 to 0.09) *P* = 0.31	0.12 (−0.06 to 0.29) *P* = .20
Time in hypoglycemia				
<3.0 mmol/L	0.13 (−0.06 to 0.30) *P* = .18	0.12 (−0.07 to 0.29) *P* = .21	0.03 (−0.15 to 0.21) *P* = .71	−0.06 (−0.23 to 0.12) *P* = .53
<3.9 mmol/L	0.09 (−0.10 to 0.27) *P* = .34	0.18 (−0.00 to 0.35) *P* = .056	0.11 (−0.07 to 0.28) *P* = .24	−0.07 (−0.24 to 0.11) *P* = .46
Time in euglycemia 3.9–8 mmol/L	0.10 (−0.09 to 0.28) *P* = .30	**0.20 (0.02 to 0.37)** ******P**** = .027**	0.10 (−0.08 to 0.28) *P* = .28	**−0.20 (−0.37 to −0.02)** ******P**** = .027**
Time in range 3.9–10 mmol/L	0.10 (−0.09 to 0.28) *P* = .32	**0.27 (0.09 to 0.43)** ***P*** **= .003**	0.04 (−0.14 to 0.22) *P* = .66	**−0.22** (**–0.38 to −0.04)** ******P**** = .017**
Time in hyperglycemia				
>10 mmol/L	−0.06 (−0.24 to 0.13) *P* = .54	−0.16 (−0.33 to 0.02) *P* = .09	−0.07 (−0.24 to 0.12) *P* = .48	**0.19 (0.01 to 0.35)** ******P**** = .040**
>13.9 mmol/L	−0.13 (−0.31 to 0.05) *P* = .17	**−0.19 (−0.36 to −0.01)** ***P*** **= .039**	−0.14 (−0.31 to 0.04) *P* = .14	−0.06 (−0.23 to 0.12) *P* = .54

Data are presented as Pearson correlation coefficient with 95% CI. Significant results are highlighted in bold.

Abbreviations: CGM, continuous glucose monitoring; CI, confidence interval; CV, coefficient of variation; DTSQc, Diabetes Treatment Satisfaction Questionnaire *change* version; DTSQs, Diabetes Treatment Satisfaction Questionnaire *status* version; HbA1c, hemoglobin A1c; MAGE, mean amplitude of glycemic excursions; Swe-PAID-20, Problem Areas in Diabetes Questionnaire, Swedish version; SD, standard deviation; SMBG, self-monitoring of blood glucose; WHO-5, World Health Organization–Five Well-Being Index.

#### Predictors of CGM effects

Evaluated baseline variables, including age, sex, diabetes duration, number of hypoglycemia events, and time in hyperglycemia, were not significantly associated with change in treatment satisfaction and well-being with CGM compared with SMBG. However, more time in hypoglycemia (<3.0 mmol/L) at baseline was associated with a lower increase in treatment satisfaction with CGM compared with SMBG in terms of DTSQ*s* (*r* = −0.22, *P* = .014) and DTSQ*c* (*r* = −0.21, *P* = .021) (Supplemental Table S3).

## Discussion

In this study from the GOLD trial, improved convenience and flexibility in use of CGM were key explanatory factors for overall improved treatment satisfaction in T1D adults managed with MDI. In contrast, perceived effects of CGM in reducing hyperglycemia and hypoglycemia were of relatively little importance for overall treatment satisfaction. Regardless of sex or age, individuals reported similar benefits from use of CGM with respect to treatment satisfaction, well-being, and emotional distress, whereas patients with more time in hypoglycemia experienced less effects on treatment satisfaction.

Researchers have reported an existing gap in knowledge regarding the experience of CGM due to a lack of comprehensive recordings of patient-reported outcomes in several studies.^17^ The current GOLD trial earlier demonstrated overall improvements in treatment satisfaction and well-being by CGM, whereas the underlying factors have not previously been explored.^8^ Similarly, in the DIAMOND trial that evaluated CGM versus SMBG in adult persons with MDI as in the current GOLD trial, increased satisfaction with CGM and well-being was also found.^18^ In the SWITCH trial, CGM showed improved treatment satisfaction in patients managed with continuous subcutaneous insulin infusion (CSII), where increased flexibility and convenience contributed.^19^ In the HypoCOMPaSS study including individuals with T1D managed with both MDI and CSII with problematic hypoglycemia, CGM showed to be more convenient and effective, and less intrusiveness regarding what patients benefit from CGM with respect to treatment satisfaction, well-being, and emotional distress.^20^

Increased flexibility and convenience explained about 60% of the overall treatment satisfaction in CGM, whereas experienced effects on hyperglycemia and hypoglycemia accounted for only 6%. Increased flexibility and convenience are likely explained by patients easily getting information on glucose levels and their directions. The fact that the patient does not need to perform capillary testing in many instances may be another important factor. As CGM in the GOLD trial earlier has been shown to reduce time in hypoglycemia (<3.9 mmol/L by around 50% and time <3.0 mmol/L by 65%), it may seem surprising that patients only experience rather small effects on hypoglycemia.^21^ One possible explanation may be that patients when using SMBG for glucose monitoring are not fully aware of their time in hypoglycemia, and thereby not either the great improvement by CGM. Correspondingly, it is difficult to explain why patients with more time in hypoglycemia experienced less effects on treatment satisfaction, in particular as we have earlier shown that this patient group has the greatest effect in reducing hypoglycemia.^22^ CGM has also earlier been shown to improve hypoglycemia confidence as well as being associated with less hypoglycemia distress.^21,23^

During CGM in the current study patients felt more cheerful, calm and relaxed, were more active, and woke up more fresh and rested. The reason why CGM affects several different fields of well-being may be due to improved flexibility and convenience with treatment and thereby influencing daily life and work. Furthermore, the improved overall glucose control, fewer hypoglycemias and less glucose variability by CGM may be other essential factors. In the current study improved HbA1c was related to overall treatment satisfaction and well-being, and more time in euglycemia and TIR with increased treatment satisfaction and less diabetes-related distress. However, reduced glucose variability by CGM was generally not related to patient-reported outcomes. Possible explanations may be that patients have over long periods in clinical practice been informed of the importance of improving HbA1c. Another factor may be that the overall control is essential for mental processes and cognitive function.^24,25^ In the DIAMOND trial comparing CGM and SMBG CGM satisfaction was unrelated to various glucose metrics.^18^

It has been debated to what extent CGM may be stressful and lead to anxiety for patients continuously getting information on their glucose levels and actions they need to take.^17^ Moreover, certain patients believe they will be stressed by carrying a device on the body. The strong data in the current study showing an overall improvement, although individual patients may have another experience, contradicts this assumption and instead shows that the convenience and flexibility are dramatically increasing with CGM. This finding is essential when considering CGM initiation for the individual patient. It is of particular importance because diabetes has been associated with burnout syndromes and depression where the complexity of treating diabetes may be one essential contributing factor.^2,25,26^ Moreover, many persons with T1D today still do not have the possibility to receive CGM. Therefore, the current findings are essential for decision makers. Continuous glucose monitoring does not only improve overall glucose control and hypoglycemias, but the treatment is also more flexible, convenient, and influences several important fields of well-being important in daily life.

A strength of the current study is a randomized design and comprehensive measures of validated patient-reported outcomes. Another strength is its relatively large size in combination with a cross-over design making it possible with intraindividual comparisons and leading to relatively high statistical power. This has been shown to be of importance in particular when it comes to evaluating patient-reported outcomes by CGM.^23^ With respect to limitations, it should be noted that patients included in the GOLD trial were Caucasian adults managed with MDI and an HbA1c of 7.5% (58 mmol/mol) or higher. Although correlations between certain glucose metrics and patient-reported outcomes existed, they were generally not strong. It is important to note that the CGM system used in the study required daily calibration and was less accurate than the generation of CGM systems currently used in clinical practice.

While CGM improves glucose control in people with T1D on MDI, increased convenience and flexibility through CGM is of even greater importance for treatment satisfaction and patient well-being. These CGM-mediated effects should be taken into account when considering CGM initiation. As women, men, younger, and older adults with various diabetes duration benefit equally by CGM, these data indicate a need in a wide group of persons with T1D. The fact that major effects on hypoglycemia by CGM seem to have relatively little effect on treatment satisfaction and well-being needs further research.

## Supplemental Material

sj-docx-1-dst-10.1177_19322968231183974 – Supplemental material for Treatment Satisfaction and Well-Being With CGM in People With T1D: An Analysis Based on the GOLD Randomized Trial Supplemental material, sj-docx-1-dst-10.1177_19322968231183974 for Treatment Satisfaction and Well-Being With CGM in People With T1D: An Analysis Based on the GOLD Randomized Trial by Daniel Pylov, William Polonsky, Henrik Imberg, Helen Holmer, Jarl Hellman, Magnus Wijkman, Jan Bolinder, Tim Heisse, Sofia Dahlqvist, Thomas Nyström, Erik Schwarz, Irl Hirsch and Marcus Lind in Journal of Diabetes Science and Technology

## References

[1] Amsberg S Wredling R Lins P-E , et al. The psychometric properties of the Swedish version of the Problem Areas in Diabetes Scale (Swe-PAID-20): scale development. Int J Nurs Stud. 2008;;45(9):1319-1328. 17983618 10.1016/j.ijnurstu.2007.09.010

[2] Rajput R Gehlawat P Gehlan D , et al. Prevalence and predictors of depression and anxiety in patients of diabetes mellitus in a tertiary care center. Ind J Endocrinol Metab. 2016;20(6):746-765. 10.4103/2230-8210.192924 PMC5105554 27867873

[3] Roy T Lloyd CE . Epidemiology of depression and diabetes: a systematic review. J Affect Disord. 2012;142(Suppl):S8-S21. doi:10.1016/S0165-0327(12)70004-6. 23062861

[4] Messer LH Weinzimer SA . Practical implementation of diabetes technology: real-world use. Diabetes Technol Ther. 2020;22:S119-S129. 10.1089/dia.2020.2509 PMC7869873 32069150

[5] Klatman E Jenkins A Ahmedani M , et al. Blood glucose meters and test strips: global market and challenges to access in low resource settings. Lancet: Diabetes Endocrinol. 2019;7(2):150-160. doi:10.1016/S2213-8587(18)30074-3. 30072234

[6] Clinton Health Access Initiative (CHAI). Market Report of Self-Monitoring Devices in LMICs; 2021. Accessed June 19, 2023. https://www.clintonhealthaccess.org/wp-content/uploads/2021/10/Market-Report_Self-monitoring-Devices-in-LMICs.pdf

[7] Lepeska M Beran D Even M , et al. Access to insulin: a comparison between low- and middle-income countries and the United Kingdom. Pract Diabetes. 2021;38(4):13-16. doi:10.1002/pdi.2345.

[8] Lind M Polonsky W Hirsch IB , et al. Continuous glucose monitoring vs conventional therapy for glycemic control in adults with type 1 diabetes treated with multiple daily insulin injections: the gold randomized clinical trial. JAMA. 2017;317:379-387. 28118454 10.1001/jama.2016.19976

[9] Lind M Polonsky W Hirsch IB , et al. Design and methods of a randomized trial of continuous glucose monitoring in persons with type 1 diabetes with impaired glycemic control treated with multiple daily insulin injections (GOLD Study). J Diabetes Sci Technol. 2016;10(3):754-761. doi:10.1177/1932296816642578. 27081191 PMC5038549

[10] Saisho Y . Use of diabetes treatment satisfaction questionnaire in diabetes care: importance of patient-reported outcomes. Int J Environ Res Public Health. 2018;15(5):947. doi:10.3390/|ijerph15050947. 29747423 PMC5981986

[11] Hajos TR Pouwer F Skovlund SE , et al. Psychometric and screening properties of the WHO-5 well-being index in adult outpatients with type 1 or type 2 diabetes mellitus. Diabet Med. 2013;30(2):e63-e69. 10.1111/dme.12040 23072401

[12] Polonsky WH Anderson BJ Lohrer PA , et al. Assessment of diabetes-related distress. Diabetes Care. 1995;18(6):754-760. 7555499 10.2337/diacare.18.6.754

[13] Welch G Weinger K Anderson B , et al. Responsiveness of the Problem Areas In Diabetes (PAID) questionnaire. Diab Med. 2003;20(1):69-72. 10.1046/j.1464-5491.2003.00832.x 12519323

[14] Bradley C . Diabetes treatment satisfaction questionnaire: change version for use alongside status version provides appropriate solution where ceiling effects occur. Diabetes Care. 1999;22:530-532. doi:10.2337/diacare.22.3.530. 10097946

[15] Grömping U . Relative importance for linear regression in R: the package relaimpo. J Stat Softw. 2006;17(1):1–27. doi:10.18637/jss.v017.i01.

[16] Grömping U . Estimators of relative importance in linear regression based on variance decomposition. Am Stat. 2007;61(2):139–147. doi:10.1198/000313007X188252.

[17] Dicembrini I Cosentino C Monami M , et al. Effects of real-time continuous glucose monitoring in type 1 diabetes: a meta-analysis of randomized controlled trials. Acta Diabetol. 2021;58(4):401-410. doi:10.1007/s00592-020-01589-3. 32789691

[18] Polonsky W Hessler D Ruedy KJ ; for the DIAMOND Study Group. The impact of continuous glucose monitoring on markers of quality of life in adults with type 1 diabetes: further findings from the DIAMOND randomized clinical trial. Diabetes Care. 2017;40(6):736-741. doi:10.2337/dc17-0133. 28389582

[19] Hommel E Olsen B Battelino T , et al; SWITCH Study Group. Impact of continuous glucose monitoring on quality of life, treatment satisfaction, and use of medical care resources: analyses from the SWITCH study. Acta Diabetol. 2014;51(5):845-851. doi:10.1007/s00592-014-0598-7. 25037251 PMC4176956

[20] Speight J Holmes-Truscott E Little SA , et al. Satisfaction with the use of different technologies for insulin delivery and glucose monitoring among adults with long-standing type 1 diabetes and problematic hypoglycemia: 2-year follow-up in the HypoCOMPaSS randomized clinical trial. Diabetes Technol Ther. 2019;21(11):619-626. doi:10.1089/dia.2019.0152. 31335201

[21] Ólafsdóttir AF Polonsky W Bolinder J , et al. A randomized clinical trial of the effect of continuous glucose monitoring on nocturnal hypoglycemia, daytime hypoglycemia, glycemic variability, and hypoglycemia confidence in persons with type 1 diabetes treated with multiple daily insulin injections (GOLD-3). Diabetes Technol Ther. 2018;20(4):274-284. doi:10.1089/dia.2017.0363. 29608107 PMC5910048

[22] Ólafsdóttir AF Bolinder J Heise T , et al. The majority of people with type 1 diabetes and multiple daily insulin injections benefit from using continuous glucose monitoring: an analysis based on the GOLD randomized trial (GOLD-5). Diabetes Obes Metab. 2021;23(2):619-630. 33200487 10.1111/dom.14257 PMC7839699

[23] Ehrmann D Heinemann L Freckmann G , et al. The effects and effect sizes of real-time continuous glucose monitoring on patient-reported outcomes: a secondary analysis of the HypoDE study. Diabetes Technol Ther. 2019;21(2):86-93. doi:10.1089/dia.2018.0332. 30615479

[24] Li W Huang E Gao S . Type 1 diabetes mellitus and cognitive impairments: a systematic review. J Alzheimers Dis. 2017;57(1):29-36. doi:10.3233/JAD-161250. 28222533

[25] Bener A Al-Hamaq AA Dafeeah E . High prevalence of depression, anxiety and stress symptoms among diabetes mellitus patients. Open Psychiatry J. 2011;5:5-12.

[26] Li C Ford ES Strine TW Mokdad AH . Prevalence of depression among U.S. adults with diabetes: findings from the 2006 behavioral risk factor surveillance system. Diabetes Care. 2008;31:105-107. 17934145 10.2337/dc07-1154

