# Supplementary material for: Treatment Satisfaction and Well-Being With Continuous Glucose Monitoring in People With Type 1 Diabetes: An Analysis Based on the GOLD Randomized Trial
Source: J Diabetes Sci Technol. 2023 Jul 27;19(1):143–52. doi: 10.1177/19322968231183974 (PMC11688678; doi:10.1177/19322968231183974)
Supplement: sj-docx-1-dst-10.1177_19322968231183974 – Supplemental material for Treatment Satisfaction and Well-Being With CGM in People With T1D: An Analysis Based on the GOLD Randomized Trial [file sj-docx-1-dst-10.1177_19322968231183974.docx]

Supplementary Appendix

This appendix has been provided by the authors to give readers additional information about their work.

Supplement to:

Treatment satisfaction and well-being with CGM in people with T1D: An Analysis Based on the GOLD Randomized Trial

**Daniel Pylov^1,2^, William Polonsky^3,4^, Henrik Imberg^5,6^, Helen Holmer^7^, Jarl Hellman^8^, Magnus Wijkman^9^, Jan Bolinder^10^, Tim Heisse^11^, Sofia Dahlqvist^12^, Thomas Nyström^13^, Erik Schwarz^14^, Irl Hirsch^15^, Marcus Lind^1,2,12^
Author Affiliations**1.Department of Molecular and Clinical Medicine, Sahlgrenska Academy, University of Gothenburg, Gothenburg, Sweden.

2.Department of medicine, Sahlgrenska University Hospital, Gothenburg, Sweden

3.Behavioral Diabetes Institute, San Diego, CA, USA;

4.University of California, San Diego, CA, USA.

5.Chalmers University of Technology and University of Gothenburg Gothenburg, Sweden.

6. Statistiska Konsultgruppen, Gothenburg, Sweden.

7.Department of Internal Medicine, Centralsjukhuset, Kristianstad, Sweden.

8 Department of Medical Sciences, Clinical Diabetes and Metabolism, Uppsala University, Uppsala, Sweden.

9. Department of Internal Medicine and Department of Health, Medicine and Caring Sciences, Linköping University, Norrköping, Sweden.

10. Department of Medicine, Karolinska University Hospital Huddinge, Karolinska Institutet, Stockholm, Sweden.

11. Profil, Neuss, Germany.

12. Department of Medicine, NU Hospital Group, Uddevalla, Sweden.

13. Department of Clinical Science and Education, Södersjukhuset, Karolinska Institutet, Stockholm, Sweden.

14. Department of Internal Medicine, Faculty of Medicine &Heatlh, Örebro University, Örebro Sweden.

15. University of Washington, School of Medicine, Seattle.

**Corresponding author: Marcus Lind, Professor of Diabetology, Institute of Medicine, University of Gothenburg, e-mail.** [**Marcus.lind@gu.se**](mailto:Marcus.lind@gu.se)

**Table of Contents**

**Table S1.** Demographics and baseline-characteristics of patients included in the current analysis and all randomized subjects…………………………………………………………………………………………...3

**Table S2**. Comparison of Diabetes Treatment Satisfaction, status version, (DTSQ*s*) between SMBG and CGM treatments......................................................……………………………………………………………...…..4

**Table S3**. Correlation between baseline variables with change in diabetes treatment satisfaction and quality of life from SBMG to CGM treatment…………………………………………………………………….…...5

**Table S1**. Demographics and baseline characteristics of patients included in the current analysis (subjects in full analysis set with available data for patient-reported outcome measures at follow-up) and all randomized subjects.

| **Variable** | **Included in current analysis**  **(n=139)** | **All randomized subjects**  **(n=161)** |
| --- | --- | --- |
| **Patient characteristics** | | |
| Age (years) | 44.6 (12.6) 44 (19; 77) | 43.7 (13.5) 44 (19; 77) |
| Female sex | 61 (43.9%) | 73 (45.3%) |
| Caucasian | 138 (99.3%) | 161 (100.0%) |
| Diabetes duration (years) | 22.1 (11.9) 22.6 (1.4; 56.6) | 22.3 (12.0) 21.5 (1.4; 57.9) |
| Current or previous smoker | 46 (33.1%) | 57 (34.4%) |
| HbA1c at inclusion (mmol/mol) | 71.8 (9.1) 70 (58; 104) | 72.7 (10.1) 71 (58; 116) |
| HbA1c at inclusion (%) | 8.72 (0.83) 8.6 (7.5; 11.7) | 8.80 (0.93) 8.65 (7.5; 12.8) |
| **Medical history** | | |
| Laser photocoagulation of the retina | 28 (20.1%) | 34 (21.1%) |
| Myocardial infarction | 3 (2.2%) | 4 (2.5%) |
| Stroke | 2 (1.4%) | 3 (1.9%) |
| Bypass-graft | 1 (0.7%) | 1 (0.6%) |
| Percutaneous coronary intervention | 2 (1.4%) | 3 (1.9%) |
| Amputation | 1 (0.7%) | 1 (0.6%) |
| Previous diabetic foot ulcer | 5 (3.6%) | 6 (3.7%) |
| Current diabetic foot (or leg) ulcer | 2 (1.4%) | 3 (1.9%) |
| Patient-reported number of hypoglycemias past two months (no/week) | 2.1 (1.9) 2 (0; 12) | 2.1 (1.8) 2.0 (0; 12) |
| Number of severe hypoglycemias past year | 0.07 (0.44) 0 (0; 3) | 0.10 (0.41) 0 (0; 3) |
| Number of severe hypoglycemias past five years | 0.6 (2.2) 0 (0; 20) | 0.7 (2.4) 0 (0; 20) |
| **Treatment satisfaction and quality of life** | | |
| Diabetes Treatment Satisfaction Questionnaire (DTSQs) total scale | 25.4 (5.8) 26.0 (4.0; 36.0) | 25.3 (5.8) 26 (4; 36) |
| WHO-5 Well-Being Index | 60.3 (17.4) 64 (12; 100) | 60.5 (17.4) 64 (12; 100) |
| Hypoglycemic Fear Scale: Behavior/Avoidance | 1.9 (0.6) 1.9 (0.6; 3.7) | 1.9 (0.6) 1.9 (0.6; 3.7) |
| Hypoglycemic Fear Scale: Worry | 0.8 (0.7) 0.8 (0; 3.6) | 0.9 (0.7) 0.8 (0.0; 3.6) |
| Problem Areas in Diabetes (Swe-PAID-20) total scale | 25.2 (17.1) 22.5 (0.0; 83.8) | 26.0 (17.8) 22.5 (0.0; 83.8) |
| Hypoglycemic Confidence Questionnaire (HCQ) total scale | 3.3 (0.5) 3.2 (2.1; 4.0) | 3.2 (0.5) 3.2 (2.1; 4.0) |
| Data are presented as mean (SD) and median (minimum value; maximum value) for numeric variables, and as number and percent for categorical variables. | | |

| **DTSQ*s* item** | **CGM  (n=131)** | **SMBG (n=131)** | **Change from SMBG to CGM** | **p-value** |
| --- | --- | --- | --- | --- |
| **Q1:** How satisfied are you with your current treatment?  (0 = very dissatisfied, 6 = very satisfied) | 5.1 (0.9) 5 (2; 6) | 4.6 (1.4) 5 (0; 6) | Decrease 23 (17.7%) Equal 51 (39.2%) Increase 56 (43.1%) | p<.0001 |
| **Q2:** How often have you felt that your blood sugars have been unacceptably high recently?  (0 = none of the time, 6 = most of the time) | 3.1 (1.2) 3 (1; 6) | 3.5 (1.4) 4 (0; 6) | Decrease 58 (44.3%) Equal 41 (31.3%) Increase 32 (24.4%) | p=0.001 |
| **Q3**: How often have you felt that your blood sugars have been unacceptably low recently?  (0 = none of the time, 6 = most of the time) | 2.3 (1.1) 2 (0; 5) | 1.9 (1.0) 2 (0; 5) | Decrease 31 (23.7%) Equal 39 (29.8%) Increase 61 (46.6%) | p=0.011 |
| **Q4**: How convenient have you been finding your treatment to be recently?  (0 = very inconvenient, 6 = very convenient) | 4.8 (1.1) 5 (1; 6) | 4.1 (1.2) 4 (0; 6) | Decrease 31 (23.7%) Equal 31 (23.7%) Increase 69 (52.7%) | p<.0001 |
| **Q5:** How flexible have you been finding your treatment to be recently?  (0 = very inflexible, 6 = very flexible) | 4.6 (1.3) 5 (1; 6) | 4.1 (1.3) 4 (0; 6) | Decrease 30 (22.9%) Equal 30 (22.9%) Increase 71 (54.2%) | p<.0001 |
| **Q6:** How satisfied are you with your understanding of your diabetes?  (0 = very dissatisfied, 6 = very satisfied) | 4.7 (1.0) 5 (1; 6) | 4.6 (1.2) 5 (0; 6) | Decrease 29 (22.3%) Equal 62 (47.7%) Increase 39 (30.0%) | p=0.32 |
| **Q7**: Would you recommend this form of treatment to someone else with your kind of diabetes?  (0 = No, I would definitely not recommend the treatment,  6 = Yes, I would definitely recommend the treatment) | 5.5 (0.8) 6 (2; 6) | 4.9 (1.3) 5 (0; 6) | Decrease 13 (9.9%) Equal 64 (48.9%) Increase 54 (41.2%) | p<.0001 |
| **Q8**: How satisfied would you be to continue with your present form of treatment?  (0 = very dissatisfied, 6 = very satisfied) | 5.3 (1.1) 6 (0; 6) | 4.5 (1.4) 5 (0; 6) | Decrease 20 (15.3%) Equal 42 (32.1%) Increase 69 (52.7%) | p<.0001 |
| Data within groups are presented as mean (SD) and median (minimum value; maximum value).  Change data are presented as number and percentage with decrease, equal or increase in scores between treatments.  Comparisons between treatments were performed using the sign test.  **Abbreviations**: CGM, continuous glucose monitoring; CI, confidence interval; DTSQs, diabetes treatment satisfaction questionnaire, status version; Q, question/item; SMBG, self-monitoring of blood glucose. | | | | |

**Table S2**. Comparison of Diabetes Treatment Satisfaction, status version, (DTSQ*s*) between SMBG and CGM treatments.

|  | **Pearson correlation coefficient (95% CI)** | | | |
| --- | --- | --- | --- | --- |
| **Variable** | **DTSQ*s*** | **DTSQ*c*** | **WHO-5** | **Swe-PAID-20** |
| **Age** | 0.13 (-0.04 to 0.29) p=0.14 | -0.15 (-0.31 to 0.02) p=0.088 | -0.04 (-0.20 to 0.13) p=0.67 | 0.14 (-0.03 to 0.30) p=0.097 |
| **HbA1c** | 0.07 (-0.10 to 0.24) p=0.41 | 0.01 (-0.15 to 0.18) p=0.87 | 0.06 (-0.11 to 0.22) p=0.50 | -0.04 (-0.21 to 0.13) p=0.64 |
| **Diabetes duration** | 0.07 (-0.10 to 0.24) p=0.41 | -0.16 (-0.32 to 0.01) p=0.058 | -0.07 (-0.23 to 0.10) p=0.42 | 0.12 (-0.04 to 0.29) p=0.14 |
| **Number of hypoglycemias past two months (patient-reported)** | -0.07 (-0.24 to 0.11) p=0.45 | 0.13 (-0.05 to 0.29) p=0.16 | -0.09 (-0.26 to 0.08) p=0.29 | 0.11 (-0.06 to 0.28) p=0.20 |
| **Number of severe hypoglycemias past year** | -0.01 (-0.18 to 0.16) p=0.90 | -0.10 (-0.26 to 0.07) p=0.25 | 0.05 (-0.12 to 0.22) p=0.54 | -0.01 (-0.17 to 0.16) p=0.93 |
| **Number of severe hypoglycemias past five years** | 0.15 (-0.02 to 0.32) p=0.079 | -0.02 (-0.19 to 0.15) p=0.78 | 0.10 (-0.07 to 0.26) p=0.25 | -0.06 (-0.22 to 0.11) p=0.50 |
| **Time in hypoglycemia** |  |  |  |  |
| <3.0 mmol/L | **-0.22 (-0.39 to -0.05) p=0.014** | **-0.21 (-0.37 to -0.03) p=0.021** | -0.07 (-0.24 to 0.11) p=0.45 | 0.00 (-0.17 to 0.18) p=0.96 |
| <3.9 mmol/L | -0.15 (-0.32 to 0.03) p=0.092 | -0.15 (-0.31 to 0.03) p=0.10 | -0.11 (-0.27 to 0.07) p=0.24 | -0.01 (-0.19 to 0.16) p=0.88 |
| **Time in euglycemia** 3.9–8 mmol/L | -0.10 (-0.27 to 0.08) p=0.30 | -0.05 (-0.23 to 0.12) p=0.54 | -0.12 (-0.29 to 0.05) p=0.17 | 0.07 (-0.11 to 0.24) p=0.46 |
| **Time in range** 3.9–10 mmol/L | -0.09 (-0.27 to 0.09) p=0.32 | -0.03 (-0.20 to 0.15) p=0.77 | -0.10 (-0.27 to 0.08) p=0.28 | 0.07 (-0.10 to 0.24) p=0.41 |
| **Time in hyperglycemia** |  |  |  |  |
| >10 mmol/L | 0.12 (-0.06 to 0.30) p=0.1813 | 0.07 (-0.11 to 0.24) p=0.4612 | 0.11 (-0.06 to 0.28) p=0.2118 | -0.06 (-0.23 to 0.12) p=0.5071 |
| >13.9 mmol/L | 0.13 (-0.05 to 0.31) p=0.14 | 0.00 (-0.18 to 0.18) p=1.00 | 0.08 (-0.09 to 0.25) p=0.37 | -0.11 (-0.28 to 0.06) p=0.21 |
| Data are presented as Pearson correlation coefficient with 95% confidence interval.  **Abbreviations**: CGM, continuous glucose monitoring; CI, confidence interval; DTSQ*c*, diabetes treatment satisfaction questionnaire, change version; DTSQ*s*, diabetes treatment satisfaction questionnaire, status version; HbA1c, hemoglobin A1c; Swe-PAID-20, problem areas in diabetes scale, Swedish version; SMBG, self-monitoring of blood glucose; WHO-5, World Health Organization-Five Well-Being Index. | | | | |

**Table S3**. Correlation between baseline variables with change in diabetes treatment satisfaction and quality of life from SBMG to CGM treatment.
